# Metabolic dysfunction-associated fatty liver disease: redefining the conceptual framework from nomenclature to emerging therapeutic paradigms

**DOI:** 10.3389/fendo.2026.1950107

**Published:** 2026-09-25

**Authors:** Rui Wang

**Affiliations:** Department of General Surgery, Affiliated Yongkang First People’s Hospital of Hangzhou Medical College, Yongkang, Zhejiang, China

**Keywords:** MASLD, nomenclature, pathogenesis, therapeutics, gut-liver axis

## Abstract

The renaming of non-alcoholic fatty liver disease (NAFLD) to metabolic dysfunction-associated fatty liver disease (MAFLD), and subsequently to metabolic dysfunction-associated steatotic liver disease (MASLD), signals a paradigm shift that repositions hepatic steatosis as a systemic metabolic disorder rather than a diagnosis by exclusion. This review synthesizes the evidence supporting the nomenclature transition, examines the pathogenic mechanisms driving disease progression, evaluates the clinical implications of the lean MAFLD phenotype, and surveys the evolving therapeutic landscape. Oxidative stress, chronic low-grade inflammation, and mitochondrial dysfunction form an interconnected network propelling the trajectory from simple steatosis to steatohepatitis and fibrosis. The bidirectional liver–adipose tissue communication axis, modulated by micronutrient homeostasis, has emerged as an integrative framework for understanding disease pathobiology. Lean MAFLD, present in 20–40% of affected individuals despite normal body mass index, challenges obesity-centered screening approaches and underscores the necessity of metabolic phenotyping in routine clinical practice. Extrahepatic complications—particularly subclinical cardiovascular injury, type 2 diabetes-associated hepatocellular carcinoma, and cardiomyopathy—remain the principal causes of death in this population. The regulatory approval of resmetirom in March 2024, together with promising phase II data on incretin-based therapies, FGF21 analogs, and PPAR modulators, has begun to reshape a therapeutic field that had long relied on lifestyle modification alone. We also discuss the gut–liver axis, genetic predisposition, economic burden, and multidisciplinary management models that together define the contemporary understanding of this disease. This review integrates these developments and identifies key gaps warranting further investigation.

## 1 Introduction

The condition now referred to as metabolic dysfunction-associated fatty liver disease has traversed a long and contested nosological path. Over four decades, what began as a histological description has evolved into one of the most extensively studied metabolic conditions of the 21st century, driven by the parallel global rise in obesity, type 2 diabetes, and sedentary lifestyles. Throughout this review, we use the term MASLD (metabolic dysfunction-associated steatotic liver disease) as the current preferred nomenclature, while retaining the historical terms NAFLD and MAFLD when describing the evolution of the field or citing studies that employed those frameworks. When Ludwig, Viggiano, McGill, and Oh introduced “non-alcoholic steatohepatitis” (NASH) in 1980, they described hepatic macrovesicular steatosis with lobular inflammation in patients whose alcohol consumption fell well below the threshold typically associated with alcoholic liver injury (1). At the time, the entity was regarded as a histological curiosity of limited clinical import. The picture has since shifted in ways the original authors could scarcely have anticipated. A 2016 meta-analysis estimated the global prevalence of NAFLD at 25–38% of the adult population, rendering it the most common chronic liver disease worldwide and an increasingly frequent indication for liver transplantation in several countries (2). By 2024, an updated analysis by Younossi and colleagues reported that the prevalence of nonalcoholic steatohepatitis (NASH) had risen to an estimated 27% among adults with NAFLD, with notable geographic variation—the Middle East and South America exceeding 30% while sub-Saharan Africa registered lower rates (3).

The gender dimension of MAFLD has received growing attention. Males exhibit higher prevalence rates of hepatic steatosis and more rapid fibrosis progression compared with females, an observation attributed in part to androgen-mediated effects on hepatic lipid metabolism and the protective role of estrogen in premenopausal women. After menopause, however, the prevalence gap narrows considerably, suggesting that hormonal status modulates disease susceptibility. Epidemiological data from the NHANES cohort have shown that postmenopausal women have a prevalence of NAFLD comparable to that of age-matched men.

What makes these numbers particularly sobering is the recognition that the leading cause of death among patients with fatty liver disease is not liver failure itself. Cardiovascular disease (CVD) accounts for approximately 40% of mortality in this population (2), positioning the condition squarely at the intersection of hepatology, endocrinology, and cardiology. Zhou et al. articulated this convergence in a 2023 international multidisciplinary consensus, emphasizing that the bidirectional relationship between MAFLD and CVD warrants routine cardiovascular risk assessment as part of standard hepatology practice (4). Beyond cardiovascular mortality, the condition also imposes a substantial economic and social toll. Allen and colleagues estimated that NAFLD-related healthcare costs in the United States alone exceed $100 billion annually when accounting for both direct medical expenditures and indirect costs related to lost productivity (5). Miao et al. projected that as MASLD prevalence continues to rise in tandem with global obesity trends, healthcare systems in low- and middle-income countries will face the greatest strain (6). These economic data strengthen the case for investing in early detection and lifestyle intervention programs. The annual per-patient cost of managing compensated cirrhosis attributable to MAFLD has been estimated at $5,000–$8,000 in the United States, rising to $30,000–$60,000 for decompensated cirrhosis and exceeding $100,000 for HCC management in the first year after diagnosis. These cost gradients powerfully illustrate the economic rationale for early intervention: preventing progression from steatosis to advanced fibrosis yields far greater cost savings than treating end-stage complications.

These epidemiological and economic realities provided the impetus for reconceptualizing fatty liver disease as a systemic metabolic disorder rather than a purely hepatic condition. The recognition that a liver-centric approach to diagnosis and management is insufficient has catalyzed a shift toward multisystem evaluation and treatment models that integrate hepatology with endocrinology, cardiology, nephrology, and primary care, consistent with the multisystem disease concept articulated by Targher and colleagues (7). The NAFLD nomenclature itself has drawn sustained criticism on several grounds. Defining a disease by what it excludes—namely, by excluding “significant” alcohol consumption—fails to capture the metabolic derangements driving pathogenesis (8, 9). The threshold for significant alcohol intake varies across guidelines, creating diagnostic inconsistency in everyday clinical practice (10, 11). Moreover, the exclusionary framework provides no mechanism to accommodate patients in whom metabolic fatty liver and alcohol-related injury coexist, a scenario encountered with considerable frequency, particularly in regions where hepatitis B virus (HBV) infection is endemic (12). Fan et al. noted that in Chinese clinical practice, MAFLD frequently coexists with both alcoholic liver disease and chronic HBV, with potential for compounded fibrosis acceleration and HCC risk (13). The management of MAFLD in HBV-endemic regions raises unique clinical questions: should antiviral therapy be prioritized over metabolic intervention, or can both be pursued simultaneously? Current evidence suggests that effective HBV suppression does not reverse MASLD-related fibrosis, as metabolic pathways operate independently of viral replication (14), supporting the need for integrated management approaches.

Yilmaz argued in a 2023 editorial that “MAFLD is the optimal definition, and MASLD is the optimal terminology,” a formulation that attempted to reconcile the conceptual merits of the positive framework with the diplomatic realities of multi-society consensus-building (9). In early 2020, an international expert panel comprising 32 specialists from 22 countries proposed the term “metabolic (dysfunction)-associated fatty liver disease” (MAFLD), accompanied by a set of positive diagnostic criteria (15). Eslam and George subsequently refined this definition in a separate international consensus published later that year (16). This proposal provoked vigorous academic debate. By 2023, the American Association for the Study of Liver Diseases (AASLD) and the European Association for the Study of the Liver (EASL) had advanced an alternative designation—”metabolic dysfunction-associated steatotic liver disease” (MASLD)—reflecting the ongoing evolution in this domain (17). A 2024 update by Rinella and colleagues detailed how the new nomenclature has been incorporated into updated AASLD practice guidance (17). Porcelli et al. reviewed the terminological evolution from NAFLD to MASLD, characterizing MASLD as a compromise intended to unify the field while preserving metabolic orientation (18), a synthesis complemented by the most comprehensive recent clinical review of MASLD in adults (19). Jiang et al., in a 2025 year-in-review article, provided a comparative update on MAFLD versus MASLD across epidemiology, mechanistic insights, and clinical trial design, concluding that the two frameworks remain complementary rather than mutually exclusive (20). The debate between MAFLD and MASLD proponents reflects deeper tensions within the hepatology community regarding the relative weight to be assigned to liver-specific versus systemic metabolic criteria in defining disease.

The present review draws on English-language literature published between 2015 and 2025 to address six interrelated questions: what evidence supports the transition from NAFLD to MAFLD terminology; how do oxidative stress, inflammation, mitochondrial dysfunction, and gut–liver axis disruption interact to drive disease progression; what clinical significance does lean MAFLD carry; what are the dominant extrahepatic complications and their underlying mechanisms; what therapeutic options have emerged in the post-resmetirom era; and what genetic and socioeconomic factors shape disease risk?

## 2 From NAFLD to MAFLD: the nomenclature transition

### 2.1 Limitations of the NAFLD framework

Three shortcomings of the NAFLD construct have emerged with increasing clarity over the past decade. The first is diagnostic inconsistency: definitions of “significant” alcohol consumption vary, with some guidelines setting thresholds at 20 g/day for women and 30 g/day for men, while others adopt 30 g/day regardless of sex (10, 11). The second involves the clinical reality of overlapping etiologies—metabolic fatty liver and alcohol-related injury frequently coexist, yet NAFLD provides no framework for accommodating this comorbidity (12). Malnick et al. noted that distinguishing purely alcoholic from purely metabolic injury is often impossible in clinical practice, as both conditions share inflammatory and fibrotic pathways (12). The third, arguably most consequential, is conceptual: the exclusionary nomenclature obscures the metabolic underpinnings of disease, potentially impairing both clinical recognition and public health awareness (8, 21). Demirtas and Yilmaz articulated this critique in a 2020 editorial, arguing that the principal pathogenic driver—metabolic dysfunction—was conspicuously absent from the disease name (8).

The stigma associated with the term “non-alcoholic” has also received attention. Younossi et al. demonstrated in a 2024 survey that the NAFLD label contributed to disease-related stigma and reduced quality of life, independent of fibrosis severity (22). Patients reported feeling blamed or judged for their condition, which in turn delayed healthcare-seeking behavior. The shift to MAFLD/MASLD terminology may partly address this concern, although whether the new terms reduce stigma in clinical encounters remains to be empirically established.

### 2.2 The MAFLD diagnostic criteria: a positive framework

The 2020 international consensus established positive diagnostic criteria that represented a departure from the exclusionary NAFLD paradigm. Diagnosis requires evidence of hepatic steatosis (confirmed by imaging, histology, or validated biomarker panels) plus at least one of three metabolic criteria: overweight or obesity (BMI ≥ 23 kg/m2 for Asian populations, ≥ 25 kg/m2 for others), type 2 diabetes mellitus (T2DM), or metabolic dysregulation defined by at least two of seven specified risk factors (15). Eslam and colleagues further clarified these criteria in a subsequent publication, specifying that the metabolic dysregulation parameters include waist circumference, blood pressure, triglycerides, HDL-C, fasting glucose, HOMA-IR, and C-reactive protein thresholds (16). A notable feature is that MAFLD does not require exclusion of alcohol consumption or coexisting viral hepatitis. The 2024 Chinese prevention and treatment guidelines adopted MAFLD as the preferred terminology, noting that it frequently coexists with both alcoholic liver disease and chronic HBV in Chinese clinical practice (13).

### 2.3 Prognostic advantages of the MAFLD framework

Several comparative studies have demonstrated that the MAFLD criteria outperform NAFLD criteria in identifying patients at greatest risk. Gofton et al. systematically compared both frameworks, concluding that the MAFLD approach better identifies individuals with elevated liver-related and extrahepatic mortality (10). Particularly compelling evidence comes from Nguyen et al., who leveraged NHANES III data to classify 2,997 individuals with ultrasonography-confirmed steatosis into three groups—NAFLD-only, MAFLD-only, and overlap. The MAFLD-only group carried the heaviest metabolic comorbidity burden: 7.9% exhibited advanced fibrosis (FIB-4 > 2.67) compared with 1.3% in the NAFLD-only group (P < 0.0001). Cumulative all-cause mortality reached 26.2% in the MAFLD-only group versus 10.6% in the NAFLD-only group (P < 0.0001), with an adjusted hazard ratio of 2.4 (95% CI: 1.2–4.6) (11). Miao et al., in a 2024 review, projected that MASLD affects over one billion adults worldwide (6).

### 2.4 Histopathological convergence with alcoholic liver disease

Malnick et al. systematically compared alcoholic liver disease (ALD) with NAFLD/MAFLD and identified a shared pathological trajectory from steatosis through inflammation and fibrosis to cirrhosis (12). The primary initiating insults differ: ALD is driven by acetaldehyde-mediated DNA damage and oxidative stress from ethanol metabolism, whereas NAFLD/MAFLD is propelled by insulin resistance and metabolic syndrome components. Gut microbiota dysbiosis, however, emerges as a common pathogenic denominator in both conditions, reinforcing the concept that metabolic perturbation is the true disease substrate (12, 23).

## 3 Pathogenic mechanisms

The pathogenesis of MASLD, summarized comprehensively in **Figure 1**, is best understood through a “multiple-hit” framework (24, 25) in which no single factor accounts for disease progression. The original “two-hit” model by Day and James (first hit = steatosis; second hit = oxidative stress/inflammation) has been progressively refined to accommodate the multiplicity of signals now recognized as pathogenic. Friedman outlined the broader mechanistic landscape in a seminal 2018 review, placing mitochondrial dysfunction within this framework alongside genetic susceptibility, gut–liver axis disruption, and inter-organ crosstalk (25).

**Figure 1:**
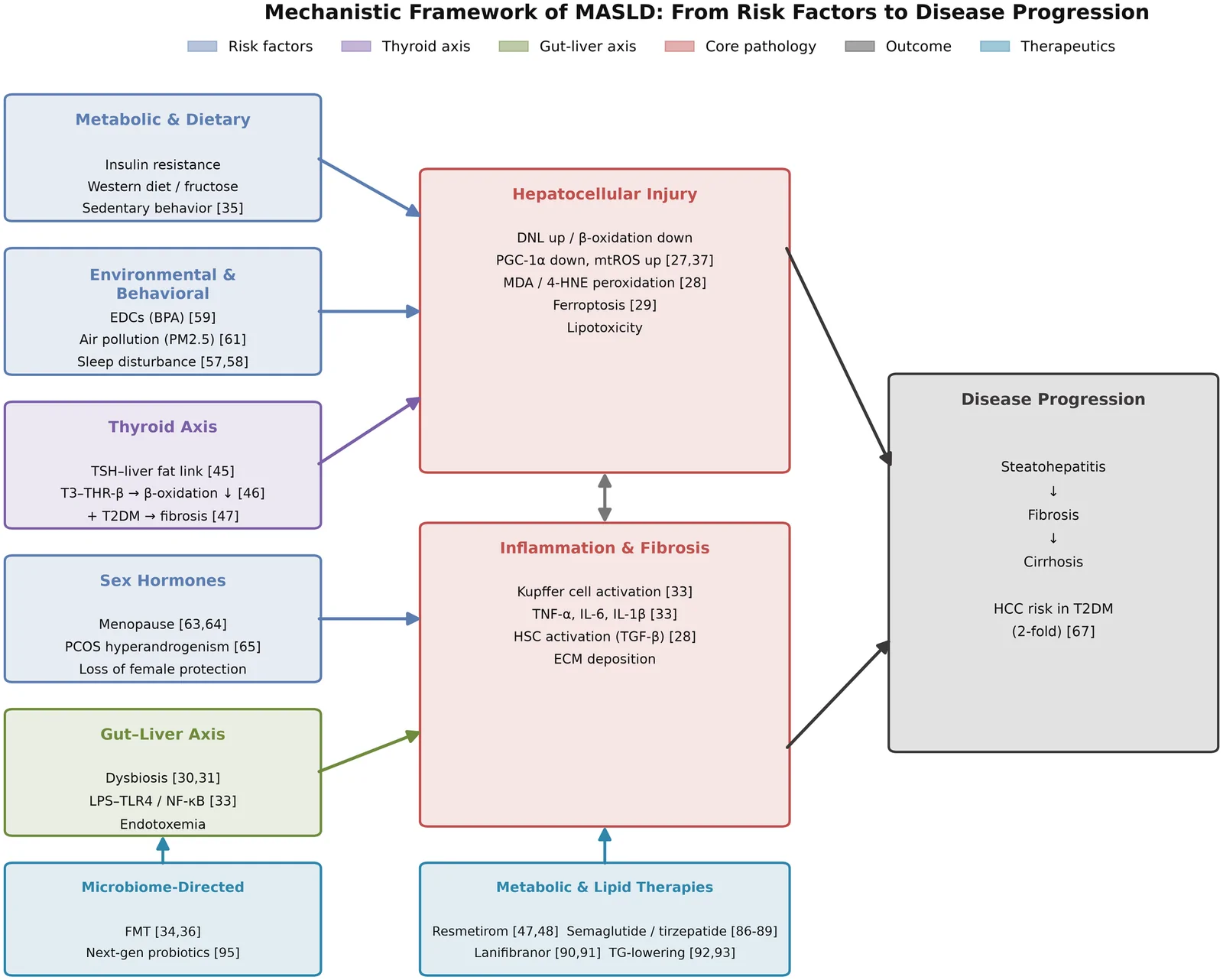
Comprehensive mechanistic framework of MASLD pathogenesis. Environmental, dietary, thyroid, and sex hormone inputs converge on insulin resistance, gut-liver axis disruption, and hepatic injury pathways, driving progression from steatosis through MASH to cirrhosis and HCC. Blue annotations indicate therapeutic targets: resmetirom (THR-beta agonist), incretin-based therapies, microbiome-directed interventions, and triglyceride-lowering strategies.

### 3.1 Insulin resistance and hepatic lipid accumulation

Insulin resistance occupies the central position in MAFLD pathogenesis. In the insulin-resistant state, adipose tissue lipolysis accelerates, flooding the portal circulation with free fatty acids (FFAs) that exceed the liver’s capacity for β-oxidation or very-low-density lipoprotein (VLDL) export. Simultaneously, hepatic *de novo* lipogenesis (DNL) becomes upregulated through the action of carbohydrate-responsive element-binding protein (ChREBP) and sterol regulatory element-binding protein 1c (SREBP-1c). Fabbrini et al. demonstrated that obese individuals with NAFLD exhibit elevated rates of DNL compared with equally obese controls without hepatic steatosis, implicating impaired hepatic insulin signaling as a specific driver rather than adiposity per se (26). The resulting lipid overload within hepatocytes initiates lipotoxicity—a state in which saturated fatty acids and ceramides activate stress kinases (JNK and IKKβ), trigger endoplasmic reticulum stress through the unfolded protein response, and set the stage for inflammatory signaling. Lipotoxicity is further amplified by impaired autophagy. In MAFLD, hepatic autophagy is suppressed by hyperinsulinemia and chronic nutrient excess, creating an additional pathway through which lipid accumulation and organelle damage propagate.

### 3.2 Oxidative stress and lipid peroxidation

Within hepatocytes, excessive FFA accumulation drives increased β-oxidation and electron transport chain activity, overproducing reactive oxygen species (ROS). When ROS generation outpaces cellular antioxidant defenses—including glutathione, superoxide dismutase, and catalase—oxidative damage to lipids, proteins, and DNA ensues (27). Lipid peroxidation products, notably malondialdehyde and 4-hydroxynonenal, inflict direct hepatocellular injury and serve as chemoattractant signals that activate hepatic stellate cells, thereby initiating fibrogenesis (28). Tauil et al. emphasized that the oxidative stress–inflammatory cascade represents the critical juncture at which simple steatosis transitions to steatohepatitis (27). More recently, Cui and Ye proposed that ferroptosis—an iron-dependent form of regulated cell death driven by lipid peroxidation—may constitute a previously underappreciated cell death modality in MAFLD, linking iron metabolism, oxidative stress, and hepatocellular injury in a unified mechanistic framework (29). Seidita et al. examined extra virgin olive oil (EVOO) as a dietary intervention rich in polyphenols capable of modulating redox homeostasis, though large-scale randomized data remain lacking (30).

### 3.3 The gut–liver axis and microbial dysbiosis

Tripathi et al. provided a comprehensive framework for understanding how the intestinal microbiome communicates with the liver through portal venous blood, bile acids, and microbial metabolites (31). Lau et al. expanded this framework in 2025, delineating how gut microbial dysbiosis contributes not only to MASLD progression but also to hepatocellular carcinogenesis through bacterial metabolite-mediated DNA damage and immune modulation (32). In MAFLD, qualitative and quantitative alterations in gut microbiota composition—collectively termed dysbiosis—increase intestinal permeability, allowing translocation of lipopolysaccharide (LPS) and other pathogen-associated molecular patterns (PAMPs) into the portal circulation. Within the liver, LPS engages Toll-like receptor 4 (TLR4) on Kupffer cells, triggering NF-κB activation and the release of pro-inflammatory cytokines including TNF-α, IL-6, and IL-1β (33). Beyond LPS, microbial-derived metabolites exert modulatory effects: trimethylamine N-oxide (TMAO) has been independently associated with cardiovascular events; secondary bile acids exhibit pro-inflammatory and pro-fibrotic properties; short-chain fatty acids generally exert beneficial metabolic effects by enhancing intestinal barrier integrity. Clinical translation of this axis has entered the trial arena: a randomized controlled trial of fecal microbiota transplantation in NAFLD demonstrated modest reductions in hepatic steatosis with donor-dependent variability (34), and a resistant-starch intervention reshaping the microbiome significantly reduced intrahepatic triglyceride content (35), establishing proof-of-concept that microbiome modulation can influence disease-relevant endpoints. Whether obese or diabetic individuals harbor a dysbiotic signature sufficiently distinct from healthy controls to serve as a therapeutic target remains an open question, though cross-sectional comparisons suggest taxonomic and functional divergence (36).

### 3.4 Inflammatory pathway activation

Chronic low-grade inflammation represents a hallmark of MAFLD. In adipose tissue, hypertrophic adipocytes and infiltrating macrophages secrete TNF-α, IL-6, and MCP-1, propagating systemic insulin resistance and hepatic inflammation (23). Within the liver, Kupffer cells activated by gut-derived LPS and damage-associated molecular patterns release additional pro-inflammatory mediators, establishing a self-amplifying loop (33). Recruitment of circulating monocytes to the inflamed liver, their differentiation into pro-fibrotic macrophages, and their cross-talk with hepatic stellate cells create a pro-fibrotic microenvironment that perpetuates fibrosis even after the initial metabolic insult is attenuated. Buzzetti et al. placed these inflammatory processes within the “multiple-hit” pathogenesis framework (24).

### 3.5 Mitochondrial dysfunction

Key manifestations of mitochondrial impairment include diminished β-oxidation capacity, reduced oxidative phosphorylation efficiency, impaired mitochondrial biogenesis (mediated by PGC-1α), and heightened ROS generation (27, 37). Lipid accumulation further compromises mitochondrial function, creating a vicious cycle. Sunny et al. provided an updated perspective, noting that early-stage disease may involve compensatory mitochondrial biogenesis that eventually becomes overwhelmed (37). Mitochondrial DNA damage—itself accelerated by ROS—impairs the transcription of electron transport chain components, creating a feed-forward cycle of organelle dysfunction and oxidative stress.

### 3.6 The liver–adipose tissue axis and micronutrient homeostasis

White adipose tissue functions as an endocrine organ whose secretome modulates hepatic lipid metabolism and insulin sensitivity (38). In obesity, adipose tissue inflammation drives increased FFA release into the portal circulation (26). Tattoli et al. examined the contribution of micronutrient deficiency to disruption of the liver–adipose axis. Vitamin D deficiency has been linked to worsened hepatic inflammation and fibrosis severity (39). Vitamin E remains one of the few agents with randomized controlled trial evidence for NASH treatment, although in the PIVENS trial fibrosis regression did not reach statistical significance versus placebo (40). Randomized trials of vitamin D supplementation in NAFLD have yielded largely null results, with meta-analyses showing modest reductions in liver enzymes but no consistent histological benefit (41); notably, these trials predominantly tested repletion of deficiency states rather than supraphysiological dosing, and adequately powered trials targeting fibrosis endpoints are lacking. Aggeletopoulou et al. provided updated mechanistic insights into the immunomodulatory and anti-fibrotic roles of vitamin D signaling through the vitamin D receptor (VDR) (42).

### 3.7 From steatosis to fibrosis: the stellate cell paradigm

The transition from simple steatosis to fibrosis is the critical determinant of clinical outcomes. Hepatic stellate cells, residing in the space of Disse in their quiescent state, serve as the principal fibrogenic effectors. Upon activation by oxidative stress, inflammatory cytokines (particularly TGF-β), and gut-derived endotoxins, stellate cells undergo phenotypic transformation into proliferative, contractile myofibroblasts that secrete excessive extracellular matrix—predominantly type I collagen (28). Friedman described this activation as a two-step process: an initiation phase driven by paracrine signals, followed by a perpetuation phase sustained by autocrine loops (28). The reversibility of early-stage fibrosis upon removal of the injurious stimulus—as demonstrated by bariatric surgery studies showing fibrosis regression after sustained weight loss (43)—highlights the dynamic nature of this process and the therapeutic window for intervention.

### 3.8 Thyroid dysfunction and MASLD

Thyroid hormone status represents an increasingly recognized modulator of hepatic lipid metabolism. A dose-response meta-analysis found that both overt and subclinical hypothyroidism were associated with increased NAFLD risk in a graded fashion (44). In a Mendelian randomization and population-based analysis published in *Gut*, primary hypothyroidism was causally linked to MASLD susceptibility (45). At the cellular level (**Figure 1**, thyroid module), T3 drives hepatic fatty acid β-oxidation and lipid clearance via thyroid hormone receptor-β (THR-β); diminished THR-β signaling reduces hepatic lipid efflux and promotes steatosis (46). Clinical data corroborate these mechanisms: in a 2025 analysis of MASLD patients, thyroid dysfunction combined with diabetes was associated with more advanced fibrosis stages than either condition alone (47). Therapeutically, this biology has been directly translated into the first approved MASH drug: resmetirom, a liver-directed THR-β agonist, improved both steatohepatitis and fibrosis in the MAESTRO program (48), validating the thyroid–liver axis as a druggable pathway rather than a bystander association. Whether screening for and correction of endogenous hypothyroidism confers histological benefit in euthyroid-range MASLD patients, however, remains untested.

### 3.9 Genetic susceptibility

The *PNPLA3* rs738409 C>G (I148M) polymorphism represents the best-characterized genetic risk factor. Liu et al. demonstrated that carriers of the GG genotype had markedly higher prevalence of hepatic steatosis and more advanced histological features (49). The *TM6SF2* E167K variant promotes hepatic fat accumulation by impairing VLDL secretion (49). Ghouse et al. reported in a 2025 genome-wide meta-analysis that nine loci are associated with higher risk of hepatocellular carcinoma development (50). These genetic variants are enriched in lean individuals with fatty liver, providing a partial explanation for why metabolic dysfunction can manifest in the absence of overweight or obesity (49, 51). Polygenic risk scores integrating multiple susceptibility variants have shown promise for identifying individuals at highest risk of progressive disease.

## 4 Lean MAFLD: a phenotype that challenges obesity-centered paradigms

### 4.1 Epidemiology and risk factors of lean MAFLD

Approximately 20–40% of individuals meeting MAFLD criteria maintain a normal body mass index—a population designated “lean MAFLD” (51) and termed the challenge of the “metabolically obese normal weight” phenotype (52). This phenotype is particularly relevant to Asian populations, where genetic predisposition, body composition differences (higher visceral-to-subcutaneous fat ratio at a given BMI), and environmental factors converge to produce fatty liver in individuals who would not meet conventional obesity thresholds. Studies from India, Pakistan, and Bangladesh have reported lean MAFLD proportions of 25–35%, driven partly by the “thin-fat” Indian phenotype. Zhu et al. identified independent risk factors including waist-to-height ratio, elevated triglycerides, and HOMA-IR (53). Body composition analysis has revealed that many lean individuals with MAFLD have disproportionately high visceral adiposity and reduced skeletal muscle mass—a phenotype sometimes termed “TOFI” (thin on the outside, fat on the inside).

The natural history of lean MAFLD remains incompletely characterized. Huo et al., in a *Gut* study drawing on three population-based prospective cohorts, reported that lean MASLD was associated with substantially elevated risks of adverse liver-related outcomes and cardiovascular mortality compared with non-lean MASLD, suggesting that the lean phenotype may carry a disproportionately adverse prognosis (54). This finding, alongside evidence that lean NAFLD independently predicts cardiovascular events (55), challenges the conventional assumption that lean fatty liver represents a milder disease variant and underscores the need for proactive metabolic phenotyping in normal-weight individuals, particularly in Asian primary care settings.

## 5 Lifestyle and environmental factors

### 5.1 Dietary patterns

Dietary composition ranks among the most modifiable determinants of MASLD risk. Western dietary patterns—characterized by high fructose intake from sugar-sweetened beverages, refined carbohydrates, and saturated fat—drive hepatic *de novo* lipogenesis and postprandial hypertriglyceridemia. Conversely, Mediterranean dietary patterns rich in monounsaturated fatty acids, polyphenols, and fiber have demonstrated consistent benefits on hepatic fat content and liver enzymes in randomized trials (56). Fructose in particular bypasses normal glycolytic regulation, flooding the DNL pathway and activating ChREBP independently of insulin, which explains its disproportionate hepat steatogenic potency relative to glucose. A landmark intervention trial demonstrated that resistant starch—a fermentable fiber reshaping the gut microbiome—significantly reduced intrahepatic triglyceride content in NAFLD patients, providing mechanistic proof that dietary modulation of the microbiome constitutes a viable therapeutic route (35).

### 5.2 Physical inactivity and sedentary behavior

Physical inactivity and prolonged sedentary time are independently associated with MASLD risk, even after adjustment for BMI. Mendelian randomization analyses suggest a causal component to this association, while observational studies indicate that breaking prolonged sitting with light activity improves postprandial glucose and triglyceride excursions (57). The mechanistic basis involves reduced skeletal muscle glucose disposal, diminished mitochondrial oxidative capacity, and decreased VLDL clearance.

### 5.3 Sleep disturbance

Short sleep duration and poor sleep quality have emerged as independent risk factors for NAFLD in multiple cohort studies (58). Proposed mechanisms include circadian disruption of hepatic lipid metabolism, altered ghrelin/leptin signaling promoting hyperphagia, increased cortisol secretion, and sympathetic activation. Sleep curtailment downregulates hepatic β-oxidation genes while upregulating lipogenic transcription factors, directly promoting steatosis independently of caloric intake.

### 5.4 Alcohol consumption below conventional thresholds

The role of alcohol consumption below conventional significance thresholds remains contested. Emerging data suggest that even light-to-moderate drinking may amplify fibrosis progression in MASLD, particularly in PNPLA3 risk-allele carriers, blurring the historical dichotomy between alcoholic and metabolic liver disease (12). This recognition has informed the MetALD designation within the 2023 multi-society nomenclature framework, acknowledging the disease spectrum occupied by combined metabolic and alcoholic etiologies.

### 5.5 Environmental exposures

Endocrine-disrupting chemicals (EDCs) including bisphenol A and phthalates activate nuclear receptors and interfere with hepatic lipid metabolism. A meta-analysis of observational studies linked EDC exposure to NAFLD risk (59), and a 2026 review synthesized mechanistic evidence for EDC-driven MASLD progression, including epigenetic reprogramming of lipogenic genes and mitochondrial toxicity (60). Air pollution adds another dimension: long-term PM2.5 exposure was associated with prevalent MAFLD in cross-sectional analyses (61), and a large prospective cohort linked ambient air pollution to incident NAFLD, with inflammation and oxidative stress as plausible mediators (62). These exposures cluster in socioeconomically disadvantaged populations, contributing to disparities in disease burden.

### 6 Sex differences in MASLD

Sex modifies both MASLD susceptibility and progression. Prevalence is higher in men before age 50, driven by visceral adiposity and androgen-mediated effects on hepatic lipid metabolism. Estrogen exerts multifaceted hepatic protection: it upregulates fatty acid oxidation, suppresses SREBP-1c-mediated lipogenesis, and attenuates inflammatory signaling (63). A dedicated 2026 review detailed sex-specific disease mechanisms, noting that natural killer T cell-mediated protection against steatohepatitis operates preferentially in females (64). After menopause, the protective advantage dissipates—postmenopausal women exhibit prevalence and fibrosis progression rates comparable to age-matched men, and hormone therapy studies suggest partial attenuation of this risk. Polycystic ovary syndrome (PCOS) exemplifies hyperandrogenism-associated liver risk: women with PCOS demonstrate markedly elevated NAFLD prevalence independent of BMI (65), and Mendelian randomization supports a causal contribution of PCOS to fatty liver disease along a “hepato-ovarian axis” (66). These sex-specific patterns carry therapeutic implications: trial populations skewed toward men may understate efficacy and safety considerations in women, and postmenopausal status merits consideration as a risk modifier in clinical risk stratification.

## 7 Disease complications: hepatic and extrahepatic

### 7.1 Hepatic complications: MASLD-associated hepatocellular carcinoma

HCC represents the most feared hepatic complication of MASLD. Yang et al. demonstrated in a meta-analysis that chronic hepatitis B or C patients with concurrent T2DM face approximately a two-fold increase in HCC risk (67). Shi et al. systematically reviewed six molecular mechanisms by which T2DM promotes hepatocarcinogenesis: hyperglycemia fostering a pro-tumorigenic microenvironment; hyperinsulinemia activating IGF-1 receptor signaling; sustained activation of NF-κB, STAT3, and JAK-STAT pathways; epigenetic dysregulation; gut microbiota dysbiosis; and activated platelets (68). Importantly, T2DM may promote HCC even in the absence of cirrhosis: Kanwal et al. reported that non-cirrhotic HCC risk was substantially elevated among patients with T2DM (HR = 3.05, 95% CI: 1.41–6.62) (69), with hepatic decompensation following a parallel trajectory among patients with coexisting T2DM (70). However, despite the elevated relative risk, the low pre-test probability of HCC in isolated non-cirrhotic MASLD means that routine HCC surveillance in this population is not recommended by current guidelines (71, 72); surveillance remains targeted at patients with cirrhosis and other high-risk categories. Li et al. reported, in an observational study, that metformin use was associated with reduced HCC incidence and mortality in patients with diabetes and chronic liver disease (73). This association, however, must be interpreted with caution: confounding by indication cannot be excluded, as patients tolerated and adhered to metformin may differ systematically from those on alternative regimens, and causation has not been established. No major hepatology or diabetes society currently recommends metformin as chemoprophylaxis for HCC (71, 74).

### 7.2 Cardiovascular disease: from risk factor to principal cause of death

Cardiovascular disease stands as the leading cause of mortality among patients with MASLD, accounting for roughly 40% of deaths (2). Mechanistically, MASLD contributes to atherosclerotic disease through several convergent pathways: insulin resistance drives atherogenic dyslipidemia with elevated triglyceride-rich lipoproteins and remnant cholesterol; systemic inflammation promotes endothelial dysfunction and arterial stiffening; and hepatic insulin resistance impairs reverse cholesterol transport. Meyersohn et al., in an analysis of 5,693 participants undergoing coronary CT angiography, demonstrated that hepatic steatosis was independently associated with major adverse cardiovascular events after adjustment for coronary artery disease severity, traditional risk factors, and statin use (75). Zhou et al. formalized this relationship in their 2023 international consensus, recommending routine cardiovascular risk assessment for all MASLD patients (4). Elbadawi et al. analyzed 7,093 NHANES III participants and found that atherogenic dyslipidemia was independently associated with subclinical myocardial injury (OR = 1.31, 95% CI: 1.14–1.52) (76).

### 7.3 MASLD-associated cardiomyopathy

Beyond atherosclerotic disease, MASLD has been linked to structural and functional cardiac abnormalities independent of traditional cardiovascular risk factors. The mechanisms underlying MASLD-associated cardiomyopathy are increasingly well delineated: ectopic cardiac lipid deposition (myocardial steatosis) impairs mitochondrial oxidative phosphorylation in cardiomyocytes; circulating ceramides and saturated fatty acids induce cardiomyocyte lipotoxicity and apoptosis; systemic inflammation activates cardiac fibroblasts, promoting interstitial fibrosis; and insulin resistance impairs myocardial glucose uptake, shifting substrate utilization toward fatty acids at higher oxygen cost. Echocardiographic studies have reported higher prevalence of left ventricular diastolic dysfunction, increased left atrial volume, and subclinical systolic impairment. Cardiac MRI-derived strain analysis has revealed subclinical left ventricular dysfunction in up to 40% of patients with NAFLD. These findings suggest that the fatty liver–failing heart axis operates through mechanisms distinct from atherosclerotic cardiovascular disease.

### 7.4 Sex hormone-binding globulin: an emerging metabolic biomarker

Gabriel-Medina et al., in a prospective study of 62 bariatric surgery patients, demonstrated that SHBG elevation at one month post-surgery independently predicted overall weight loss at 24 months (OR = 2.71, 95% CI: 1.11–6.60) (77). Given that bariatric surgery can resolve NASH and regress fibrosis (43), SHBG may offer utility in postoperative metabolic monitoring and potentially as a non-invasive marker of treatment response in pharmacological trials.

### 7.5 Chronic kidney disease and MASLD

Kueh et al. conducted a global meta-analysis demonstrating that the coexistence of MASLD and CKD is associated with significantly higher risks of all-cause mortality and cardiovascular events compared with either condition alone (78). Mechanistically, the kidney injury in MASLD mirrors hepatic pathogenesis: insulin resistance drives glomerular hyperfiltration and podocyte injury; systemic inflammation activates renal tubulointerstitial fibrotic pathways; dyslipidemia promotes renal arteriolar sclerosis; and activation of the renin–angiotensin–aldosterone system amplifies both metabolic and hemodynamic injury. These shared pathways explain why MASLD severity correlates with CKD progression rate, and why therapeutic strategies targeting insulin resistance may confer renoprotection.

## 8 Clinical management: from risk stratification to emerging pharmacotherapy

### 8.1 Risk stratification and fibrosis assessment

Both Eastern and Western guidelines endorse targeted screening of high-risk populations (13, 79). The Fibrosis-4 (FIB-4) index offers a practical first-line tool; values below 1.30 generally rule out advanced fibrosis, while values exceeding 2.67 warrant further evaluation. Enhanced liver fibrosis (ELF) test and vibration-controlled transient elastography (FibroScan) provide incremental diagnostic accuracy, with the latter achieving ROC AUC values exceeding 0.85 for F3–F4 fibrosis detection (13, 80).

### 8.2 Approved and established therapies

Two tiers of therapy now exist: established interventions with long-standing evidence, and regulatory-approved pharmacotherapy.

#### 8.2.1 Lifestyle intervention

Vilar-Gomez et al. demonstrated that weight reduction of ≥ 7% was associated with histological NASH improvement, while ≥ 10% was required for fibrosis regression (81). Sangouni et al. found that Mediterranean dietary patterns significantly reduced liver enzyme levels in randomized controlled trials (56). A 2025 systematic review confirmed that structured dietary programs combined with 150–200 minutes of moderate-intensity exercise per week yield the most consistent histological improvements (82). Resistant-starch supplementation represents a microbiome-directed dietary strategy with demonstrated intrahepatic triglyceride reduction (35).

#### 8.2.2 Established pharmacotherapy

Vitamin E (800 IU/day) improved steatohepatitis in the PIVENS trial, though fibrosis regression did not reach statistical significance versus placebo and long-term safety concerns have restricted its adoption (40). Pioglitazone (30 mg/day) improved steatohepatitis and fibrosis but is associated with weight gain and fluid retention (40, 83).

#### 8.2.3 Regulatory-approved therapy: resmetirom

The approval of resmetirom by the FDA in March 2024 represented a watershed moment—the first pharmacotherapy approved for MASH with fibrosis. In the phase III MAESTRO-NASH-1 trial, resmetirom achieved MASH resolution without worsening fibrosis (25.9% for 80 mg vs. 11.1% for placebo, P < 0.001) and fibrosis improvement without worsening MASH (23.7% vs. 11.8%, P = 0.001) (48). Resmetirom is a liver-directed selective THR-β agonist that enhances hepatic lipid metabolism and reduces lipotoxicity (46). In August 2025, the European Commission granted conditional marketing authorization. Exploratory quantitative histomorphometry (qFibrosis) analyses further support quantitative fibrosis regression with resmetirom (84). Do et al., in a 2025 Nature Reviews Drug Discovery analysis, mapped the therapeutic landscape of MASH across more than 150 clinical-stage compounds, categorizing them by mechanistic target and trial phase (85).

### 8.3 Emerging therapeutic strategies

#### 8.3.1 Incretin-based therapies

The incretin field has advanced decisively into phase III. Semaglutide 2.4 mg achieved NASH resolution in 59% of patients in a phase II trial, though fibrosis regression did not reach statistical significance (86). The subsequent ESSENCE phase III trial (published in the *New England Journal of Medicine*, 2025) randomized 1,197 patients with MASH and F2–F3 fibrosis and met both primary endpoints: semaglutide significantly improved steatohepatitis and fibrosis compared with placebo (87), with subsequent analyses delineating weight-dependent and weight-independent hepatic effects (88). Tirzepatide has shown encouraging phase II results on steatohepatitis and fibrosis (89). These agents occupy a distinct mechanistic niche from resmetirom—addressing systemic metabolic dysfunction rather than hepatic lipid clearance alone—raising the prospect of combination therapy, which remains to be formally tested.

#### 8.3.2 Fibroblast growth factor 21 analogs

Pegbelfermin showed fibrosis improvement in the FALCON 2 trial, though primary endpoint results were mixed (90). Jeong et al. conducted a systematic review of FGF21 analogs, concluding that effects on fibrosis endpoints remained uncertain (91).

#### 8.3.3 Pan-PPAR agonism

Lanifibranor, a pan-PPAR agonist, improved insulin resistance and hepatic steatosis in phase II development, and its phase III program is ongoing (92).

#### 8.3.4 Triglyceride-lowering therapies

An emerging therapeutic avenue targets triglyceride metabolism directly. A recent comprehensive review synthesized mechanisms, efficacy, and clinical perspectives of triglyceride-lowering approaches to hepatic steatosis, spanning omega-3 fatty acids, apolipoprotein C-III antisense (volanesorsen), and angiopoietin-like protein 3 inhibition (93). The rationale is grounded in triglyceride-rich lipoprotein metabolism: reducing hepatic VLDL triglyceride availability and circulating remnant cholesterol addresses both steatosis and the atherogenic dyslipidemia linking MASLD to cardiovascular risk (94). The convergence of hepatic and cardiovascular benefit distinguishes this strategy from liver-directed agents and positions triglyceride lowering as a candidate component of combination regimens for patients with concomitant dyslipidemia, although dedicated outcome trials in MASH populations are awaited.

### 8.4 The role of bariatric surgery

Lassailly et al. demonstrated in a prospective study with 5-year follow-up that bariatric surgery resolved NASH in 84% of patients and reversed fibrosis in 70% (43). Gabriel-Medina et al. showed that early metabolic recovery markers—particularly SHBG elevation—can predict long-term weight loss outcomes (77). However, bariatric surgery carries risks including nutritional deficiencies and potential perioperative hepatic complications.

### 8.5 Economic burden and healthcare implications

Allen et al. provided a global framework for estimating the healthcare and socioeconomic costs of NAFLD (5). Miao et al. projected that as MASLD prevalence continues to rise, healthcare systems in low- and middle-income countries will face the greatest strain (6). These economic data strengthen the case for investing in early detection and lifestyle intervention programs.

### 8.6 Microbiome-based therapies: FMT and next-generation probiotics

Microbiome-directed interventions constitute a frontier strategy grounded in the gut–liver axis. A randomized controlled trial of fecal microbiota transplantation (FMT) in NAFLD demonstrated modest reductions in hepatic steatosis, with efficacy contingent on donor selection—lean, metabolically healthy donors conferred greater benefit (34). A 2025 study of consecutive FMT in MASLD reported improvements in metabolic parameters and hepatic steatosis, though durability beyond the treatment period remains to be established (95). Next-generation probiotics represent a refinement of this concept: *Akkermansia muciniphila*, a mucin-degrading bacterium enriched in metabolically healthy individuals, has demonstrated metabolic benefits in early-phase human studies, and engineered strains with enhanced therapeutic payloads are in preclinical development (36). Synbiotic combinations have yielded positive results on liver enzymes and inflammatory markers in randomized trials. Safety considerations—including transmission risk, long-term microbial stability, and regulatory classification—currently constrain clinical deployment, and no microbiome-based therapy has yet received regulatory approval for MASH. Nevertheless, the microbiome remains among the most mechanistically rational therapeutic targets, given its centrality to bile acid metabolism, endotoxin exposure, and short-chain fatty acid production (36).

### 8.7 Multidisciplinary care models

The Chinese 2024 guidelines call for joint management by hepatology, endocrinology, cardiology, and nutrition departments (13). Telemedicine and digital health platforms have emerged as potentially valuable tools for delivering multidisciplinary MAFLD care, particularly in resource-limited settings.

## 9 Discussion

Stepping back from the individual lines of evidence surveyed above, three observations warrant emphasis. First, the MAFLD criteria are not merely a relabeling exercise; they function as a prognostic enrichment tool. When Nguyen et al. reclassified NHANES III participants using both frameworks side by side, the group captured exclusively by MAFLD—missed by NAFLD—displayed a fibrosis prevalence six times higher and an all-cause mortality 2.4-fold greater than the NAFLD-only subset (11). This differential is not a statistical artifact; it reflects the fundamental insight that metabolic dysfunction, not alcohol abstinence, determines who is most likely to progress to cirrhosis and death.

Second, the therapeutic landscape has shifted from a desert to a nascent orchard. Resmetirom’s dual histological efficacy—simultaneously resolving MASH and improving fibrosis in a registration trial (48)—would have seemed implausible five years ago. Yet this single agent leaves critical questions unanswered. The MAESTRO population was carefully selected; whether comparable efficacy translates to patients with portal hypertension, decompensated cirrhosis, or concurrent HBV—the very patients most in need—remains undetermined. The drug’s mechanism, hepatic THR-β agonism, addresses lipid substrate overload but does not directly target fibrogenesis or gut-derived inflammation. This gap raises the question of whether resmetirom will serve as monotherapy or as a backbone for combination regimens. Pairing a THR-β agonist with an incretin-based agent—the former reducing hepatocellular lipotoxicity, the latter addressing systemic metabolic dysfunction and producing substantial weight loss—represents a rational, if untested, strategy.

Third, the field’s understanding of who is at risk remains incomplete in a clinically consequential way. Lean MAFLD exemplifies this gap. The finding by Huo et al. that normal-weight MASLD patients face disproportionately adverse outcomes (54) should provoke a reckoning with screening guidelines that remain anchored in BMI. In Asian clinical settings, where 30–40% of MAFLD patients have normal BMI, the current approach—selectively screening overweight individuals—systematically overlooks a high-risk population. A pragmatic alternative would integrate metabolic markers (HOMA-IR, triglyceride-glucose index, waist-to-height ratio) into primary care screening bundles, though prospective validation in multi-ethnic cohorts is needed before guideline adoption.

The gut–liver axis illustrates a different kind of uncertainty. Tripathi et al. identified microbial compositional shifts correlating with disease severity (31), but the leap from correlation to causation has not been made. Functional redundancy within the gut microbiome means that taxonomic signatures detected by 16S rRNA sequencing may not map onto meaningful differences in metabolite production. Metagenomic and metabolomic profiling, rather than compositional taxonomy alone, will be necessary to identify druggable microbial targets. This distinction matters beyond academic curiosity: the microbiome modulates the pharmacokinetics of resmetirom, pioglitazone, and likely other agents, meaning that microbial variability could contribute to inter-individual differences in drug response—a dimension of precision therapeutics that remains largely unexplored in hepatology.

Micronutrient deficiency occupies a similarly liminal position. Observational data consistently link vitamin D insufficiency to more severe fibrosis (39, 42), and the PIVENS trial established vitamin E’s modest histological benefit (40). But observational associations are vulnerable to confounding by diet, physical activity, and socioeconomic status. What is needed are biomarker-stratified randomized trials: enrolling patients with documented deficiency, targeting specific micronutrient combinations, and powered for fibrosis endpoints rather than steatosis alone. Such trials, if positive, could identify low-cost interventions suitable for resource-constrained settings where resmetirom and incretin agonists may be financially out of reach.

From a health systems perspective, the observation that cardiovascular disease and cancer—not liver failure—drive mortality in this population has profound implications for care delivery. The cardiologist prescribing a statin, the endocrinologist initiating a GLP-1 agonist, and the hepatologist dispensing resmetirom are each addressing facets of the same metabolic syndrome, yet they often operate in silos with limited communication. Breaking down these barriers will require new models of shared decision-making, potentially supported by digital health platforms that track metabolic parameters in real time.

Finally, the economic calculus is stark. The cost differential between managing simple steatosis and treating decompensated cirrhosis or HCC spans nearly an order of magnitude (5). As Miao et al. projected, the rising global MASLD burden will disproportionately strain healthcare systems in low- and middle-income countries (6), where the per-patient cost of newly approved pharmacotherapies may exceed annual per capita healthcare expenditure. This reality argues for sustained investment in primary prevention as the most cost-effective strategy for reducing the long-term burden of MAFLD and its extrahepatic complications.

## 10 Knowledge gaps and future directions

Despite two decades of intensive research, several gaps constrain both mechanistic understanding and clinical management of MASLD.

### 10.1 Prospective characterization of lean MASLD

The adverse prognosis of lean MASLD rests largely on observational cohorts subject to reverse causation and residual confounding. Prospective, multi-ethnic longitudinal studies with standardized metabolic phenotyping—incorporating body composition analysis, genetic risk scores, and insulin sensitivity indices—are needed to establish whether the lean phenotype carries independent prognostic weight or serves as a marker of distinct pathophysiology requiring tailored surveillance.

### 10.2 Causal dissection of the microbiome

Microbial associations with disease severity remain correlational. What the field requires are intervention studies—FMT, next-generation probiotics, and dietary microbiome modulation—with histological endpoints, alongside mechanistic gnotobiotic experiments establishing which microbial functions, rather than taxa, drive disease. The pharmacomicrobiomics of MASLD therapy (how the microbiome alters drug response) is essentially uncharted.

### 10.3 Optimal combination pharmacotherapy

With resmetirom approved and incretin-based therapies advancing through phase III, the sequential question—which agent for which patient, and in what combination—has no evidence base. Factorial trials pairing THR-β agonism with incretin therapy, and triglyceride-lowering agents with antifibrotics, are logical next steps. Biomarkers predicting response to each mechanism remain unvalidated.

### 10.4 Environmental epidemiology

The contributions of EDCs, air pollution, and sleep disruption are supported by observational data but lack intervention evidence. Whether exposure reduction modifies disease trajectories is untested—a gap with particular relevance for policy given the socioeconomically patterned distribution of these exposures.

### 10.5 Pediatric MASLD

Disease arising in children follows distinct trajectories, yet most mechanistic and therapeutic evidence derives from adult populations. Trials incorporating pediatric populations and studies of disease origins—including *in utero* and early-life exposures—remain scarce relative to the growing pediatric burden.

### 10.6 Implementation science

Effective lifestyle interventions exist but fail at scale. Real-world data on digital health delivery, task-shifting to primary care, and cost-effectiveness of early detection programs in diverse health systems will determine whether therapeutic advances translate into population-level impact.

## 11 Conclusions

The transition from NAFLD to MAFLD and MASLD nomenclature represents far more than semantic housekeeping: it codifies the reconceptualization of fatty liver disease as a systemic metabolic disorder and, as comparative outcome data demonstrate, identifies a higher-risk population than the framework it replaced. The pathogenesis involves a complex interplay of insulin resistance, oxidative stress, inflammatory signaling, mitochondrial dysfunction, and thyroid axis modulation, further shaped by gut–liver axis disruption, genetic susceptibility, environmental exposures, sex-specific biology, and micronutrient status. Lean MASLD challenges obesity-centered screening and necessitates broader metabolic phenotyping. Hepatic and extrahepatic complications—notably hepatocellular carcinoma and cardiovascular disease—remain the principal determinants of patient outcomes. The therapeutic landscape has been permanently altered by resmetirom’s approval, with incretin-based therapies reporting positive phase III results, and FGF21 analogs, pan-PPAR agonists, triglyceride-lowering strategies, and microbiome-directed interventions advancing through development. The challenges ahead lie in matching mechanistic heterogeneity with therapeutic precision: validating biomarkers that predict treatment response, establishing rational combination regimens, addressing the environmental and socioeconomic determinants of disease, and ensuring that therapeutic progress reaches the populations bearing the greatest burden.

## Glossary

ALD: alcoholic liver disease
AASLD: American Association for the Study of Liver Diseases
BMI: body mass index
ChREBP: carbohydrate-responsive element-binding protein
CVD: cardiovascular disease
DNL: *de novo* lipogenesis
EASL: European Association for the Study of the Liver
ELF: enhanced liver fibrosis
FFA: free fatty acid
FGF21: fibroblast growth factor 21
FIB-4: Fibrosis-4 index
GLP-1: glucagon-like peptide-1
HCC: hepatocellular carcinoma
HBV: hepatitis B virus
HOMA-IR: homeostatic model assessment of insulin resistance
HR: hazard ratio
IL-6: interleukin-6
JAK-STAT: Janus kinase-signal transducer and activator of transcription
LPS: lipopolysaccharide
MAFLD: metabolic dysfunction-associated fatty liver disease
MASLD: metabolic dysfunction-associated steatotic liver disease
MASH: metabolic dysfunction-associated steatohepatitis
NAFLD: non-alcoholic fatty liver disease
NASH: non-alcoholic steatohepatitis
OR: odds ratio
PGC-1α: peroxisome proliferator-activated receptor gamma coactivator 1-alpha
PNPLA3: patatin-like phospholipase domain-containing protein 3
PPAR: peroxisome proliferator-activated receptor
ROS: reactive oxygen species
SREBP-1c: sterol regulatory element-binding protein 1c
SHBG: sex hormone-binding globulin
T2DM: type 2 diabetes mellitus
TMAO: trimethylamine N-oxide
TNF-α: tumor necrosis factor-α
THR-β: thyroid hormone receptor-β
TLR4: Toll-like receptor 4
VDR: vitamin D receptor
VLDL: very-low-density lipoprotein
